# The clinical use of biomarkers as prognostic factors in Ewing sarcoma

**DOI:** 10.1186/2045-3329-2-7

**Published:** 2012-02-08

**Authors:** Annmeik M van Maldegem, Pancras CW Hogendoorn, Andrew B Hassan

**Affiliations:** 1 Department of Oncology University of Oxford, Oxford, OX3 7LJ, UK; 2 Sir William Dunn School of Pathology, University of Oxford, Oxford, OX1 3RE, UK; 3 Department of Pathology, Leiden University Medical Centre, PO Box 9600, 2600 RC Leiden, The Netherlands

**Keywords:** Ewing sarcoma, prognostic, biomarkers

## Abstract

Ewing Sarcoma is the second most common primary bone sarcoma with 900 new diagnoses per year in Europe (EU27). It has a poor survival rate in the face of metastatic disease, with no more than 10% survival of the 35% who develop recurrence. Despite the remaining majority having localised disease, approximately 30% still relapse and die despite salvage therapies. Prognostic factors may identify patients at higher risk that might require differential therapeutic interventions. Aside from phenotypic features, quantitative biomarkers based on biological measurements may help identify tumours that are more aggressive. We audited the research which has been done to identify prognostic biomarkers for Ewing sarcoma in the past 15 years. We identified 86 articles were identified using defined search criteria. A total of 11,625 patients were reported, although this number reflects reanalysis of several cohorts. For phenotypic markers, independent reports suggest that tumour size > 8 cm and the presence of metastasis appeared strong predictors of negative outcome. Good histological response (necrosis > 90%) after treatment appeared a significant predictor for a positive outcome. However, data proposing biological biomarkers for practical clinical use remain un-validated with only one secondary report published. Our recommendation is that we can stratify patients according to their stage and using the phenotypic features of metastases, tumour size and histological response. For biological biomarkers, we suggest a number of validating studies including markers for 9p21 locus, heat shock proteins, telomerase related markers, interleukins, tumour necrosis factors, VEGF pathway, lymphocyte count, and a number of other markers including Ki-67.

## Introduction

Ewing sarcoma is the second most common primary bone sarcoma. It is an orphan state disease with approximately 900 new diagnoses a year in Europe [1]. It is also called the Ewing Sarcoma Family of Tumours (ESFT) and includes Ewing sarcoma of bone, extra-osseous Ewing sarcoma, Primitive Neuroectodermal (PNET) and Askin's tumours. Ewing sarcoma is diagnostically defined by a Ewing sarcoma EWS (chromosome 22) translocation resulting in fusion with an ETS transcription factor, the commonest abnormality (85%) being EWS-FLI1 (chromosome 11). Ewing sarcoma is a disease affecting children and young adults with a peak incidence at age fifteen. With current treatment options the 5 year survival for non-metastatic disease is 60-70%. However, survival for the 25% of patients that present with metastatic disease is approximately 20% [2], and for those who develop relapsed and/or refractory disease, the survival is no more than 10%.

Current patients are subdivided by disease stage, namely non-metastatic, metastatic and recurrence, and patients in each group are treated the same. But apparently this subdivision is not always related to clinical outcome, because of the patients who present with non-metastatic disease, approximately 30% die within 5 years. This group may be currently undertreated while the 70% who survive may be over-treated. It may therefore be important to separate the high risk patients from the low risk patients and to be able to detect chemotherapy resistance and metastases early.

A way of predicting patients' outcome is by using prognostic factors. The most commonly used are clinical features, eg age, gender, metastases. Biomarker is a synonym for biological markers and is defined as "a characteristic that is objectively measured and evaluated as an indicator of normal biological processes, pathogenic processor or pharmacologic responses to a therapeutic intervention" [3]. Biomarkers are currently already being used for screening, diagnosis, prognosis and monitoring of cancer patients. In 2005 the Reporting recommendations for tumour MARKer prognostic studies (REMARK) guidelines were published [4]. The goal of these guidelines is to make the results from clinical prognostic studies transparent and to improve the level of comparison that is possible between studies.

We report an overview of the research which has been done to identify reliable biomarkers for Ewing sarcoma in the past 15 years, where we detail the kind of markers that have been tested, the number of patients involved and the p-value showing the significance of the marker. The results highlight some interesting biomarkers, but they have yet to be validated.

## Materials and methods

### Search strategy

We report data available in the public domain only. Papers were identified from PubMed searches and from references in the found articles. The search algorithm was: (Ewing sarcoma) AND (prognostic factors) OR (biomarker). Only papers published between 1995 and 2010 are included. The latest search was done in June 2010. Whenever multiple reports from the same study were published, we used only the report with the latest published date to avoid any duplication of information. Papers were eligible if they: (1) described (or cited a paper that described) a Ewing sarcoma study of prognostic factors or biomarkers; (2) were published in English; and (3) came from industrialized countries. All types of evaluation were accepted (full papers, conference abstracts, reports) as long as results (including data) were presented.

### Data extraction

Data extraction was conducted independently by the first author (A.M. v. M.). We used a systematic method for the search normally used for meta-analysis [5]. Differences in data extraction were resolved by consensus with a second author (A.B.H). From each eligible trial we recorded authors' names, journal and year of publication and the results from the study.

## Results and Discussion

### Eligible trials

**Figure 1:**
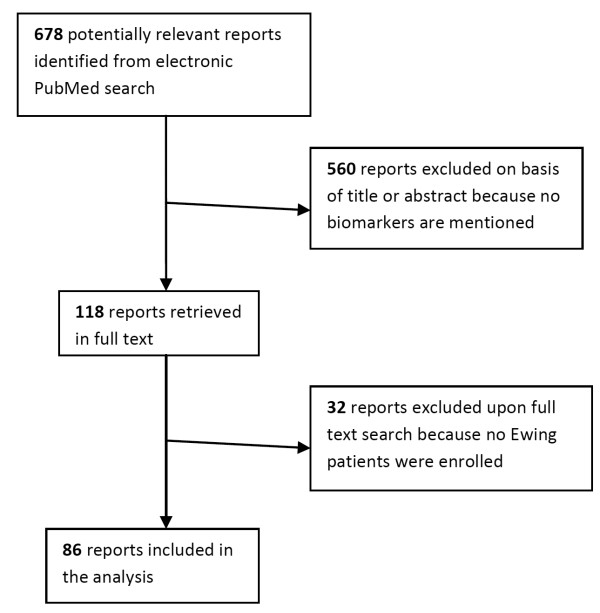
**Flowchart for the identification of eligible reports**.

A flow-chart indicating the identification of reports for inclusion in the analysis is reported for Ewing sarcoma (Figure 1). During the search many reports had to be excluded mainly because no prognostic markers were reported in the article. When we searched the reports using full text, we had to exclude some papers because no Ewing sarcoma patients were included in these reports. We identified 86 articles which were eligible for our search criteria. In these papers a total of 11, 625 patients were reported.

**Figure 2:**
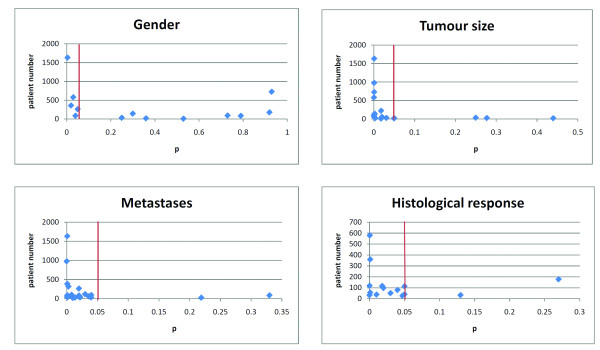
**Distribution of p related to patient number for the phenotypic markers: gender, tumour size, metastases and histological response**. The red line shows the cut-off point of p = 0.05.

In this report we looked at the published data on the use of biomarkers for the last 15 years. Biomarkers were grouped into phenotypic markers and biological markers. Markers were taken as statistically significant if p < 0.05. For phenotypic markers we reported the outcome for gender, tumour size, presence of metastases and histological response after treatment (Tables 1, 2, 3 &4). We showed the p-value reported in the eligible articles and the distribution of p correlated to the number of patients (Figures 2). There doesn't seem to be a relationship between the number of patients and the p-value. For example, the distribution of histological response shows that the studies with small patient numbers have the same statistical significance as these with large patient numbers. Throughout this report, the assumption is that the biomarker has a linear relationship to outcome. We know that for many biomarkers, this is not the case. For example, data transformation using either bicubic splines or fractional polynomials is often required to correlate continuous relationships between biomarkers and outcome, as opposed to predefined cutpoints [6]. We can only have limited extrapolation of the reported data to outcome as in most instances these questions have not been addressed.

**Table 1:** Outcome for phenotypic marker: gender

Author	Year	Pt number	P
Craft et al, Eur J Cancer 33 (7), 1061-9[8]	1997	142	0.3

Aparicio et al, Oncology 55, 20-6 [9]	1998	116	NS

Ahrens et al, Med Pediatr Oncol 32, 186-95 [10]	1999	177	0.92

Ginsberg et al, J Clin Oncol 17, 1809-14[11]	1999	85	0.79

Givens et al, Int J Oncol 14 (6), 1039-43[12]	1999	85	NS

Bacci et al, J Clin Oncol 18, 4-11[13]	2000	359	0.02

Jenkin et al, Med Pediatr Oncol 37, 383-9[14]	2001	93	0.73

Krasin et al, Cancer 104, 367-73[15]	2005	33	0.25

Bacci et al, Acta Oncol 45, 469-75[16]	2006	579	0.03

De Angulo et al, J Pediatr Hematol Oncol 29 (1), 48-52[17]	2007	24	NS

Leavey et al, Pediatr Blood Cancer 51 (3), 334-8[18]	2008	262	0.05

Jawad et al, Cancer 115, 3526-36[19]	2009	1631	0.004

Kikuta et al, Clin Cancer Res 15 (8), 2885-94[20]	2009	8	0.53

Sari et al, Pediatr Blood Cancer 54, 19-24[21]	2010	87	0.04

Xie et al, Chin J Cancer 29 (4), 420-4	2010	18	0.36

NS: not significant

**Table 2:** Outcome for phenotypic marker: tumour size

Author	Year	Pt number	P
Aparicio et al, Oncology 55, 20-6[9]	1998	116	0.0016

Kawai et al, Cancer 82, 851-9[22]	1998	20	0.0038

Ahmad et al, Cancer 85, 725-31[23]	1999	24	0.277

Givens et al Int J Oncol 14 (6), 1039-43[12]	1999	85	NS

Cotterill et al, J Clin Oncol 18, 3108-14[24]	2000	975	0.001

De Alava et al, Cancer 89, 783-92[25]	2000	55	0.02

Jenkin et al, Med Pediatr Oncol 37, 383-9[14]	2001	93	0.0001

Oberlin et al, B J Cancer 85 (11), 1646-54[26]	2001	141	0.002

Rutkowski et al, J Surg Oncol 84, 151-9[27]	2003	13	0.05

Krasin et al, Pediatr Blood Cancer 43, 229-36[28]	2004	37	S

Matsunobu et al, Clin Cancer Res 10, 1003-12[29]	2004	21	0.05

Krasin et al, Cancer 104, 367-73[28]	2005	33	0.25

Aksnes et al, Acta Oncol 45, 38-46[30]	2006	56	0.001

Bacci et al, Acta Oncol 45, 469-75[16]	2006	579	0.0004

Mikulic et al, J Pediatr Surg 41, 524-9[31]	2006	27	0.031

Cheung et al, Clin Cancer Res 13 (23), 6978-83[32]	2007	28	NS

Rodriguez-Galindo et al, Ann Oncol 19, 814-20[33]	2008	220	0.018

Yonemori et al, J Cancer Res Clin Oncol 134, 389-95[34]	2008	79	S

Jawad et al, Cancer 115, 3526-36[19]	2009	1631	0.001

Kikuta et al, Clin Cancer Res 15 (8), 2885-94[20]	2009	8	0.018

Lee et al, Cancer 116, 1964-73[35]	2010	725	0.001

Xie et al, Chin J Cancer 29 (4), 420-4	2010	18	0.44

NS: not significant, S: significant

**Table 3:** Outcome for phenotypic marker: metastases

Author	Year	Pt number	P
Terrier et al, Eur J Cancer 31 (3), 307-14[36]	1995	315	0.003

Terrier et al, Semin Diagn Pathol 13 (3), 250-7[37]	1996	315	S

Aparicio et al, Oncology 55, 20-6[9]	1998	116	0.03

De Alava et al, J Clin Oncol 16 (4), 1248-55[38]	1998	99	0.008

Paulussen et al, J Clin oncol 16 99), 3044-52[39]	1998	114	S

Ahmad et al, Cancer 85, 725-31[23]	1999	24	0.219

Baldini et al, Ann Surg 230 (1), 79-86[40]	1999	37	0.002

Ginsberg et al, J Clin Oncol 17, 1809-14[11]	1999	85	0.33

Luksch et al, Tumori 85 (2), 101-7[41]	1999	73	S

Cotterill et al, J Clin Oncol 18, 3108-14[24]	2000	975	0.0001

De Alava et al, Cancer 89, 783-92[25]	2000	55	0.02

Wei et al, Cancer 89, 793-9[42]	2000	39	0.001

Jenkin et al, Med Pediatr Oncol 37, 383-9[14]	2001	93	0.04

Zielenska et al, Cancer 91, 2156-64[43]	2001	26	0.0137

Martin et al, Arch Surg 138, 281-5[44]	2003	59	0.02

Fuchs et al, Clin Cancer Res 10, 1344-53[45]	2004	31	0.022

Matsunobu et al, Clin Cancer Res 10, 1003-12[29]	2004	21	NS

Weston et al, B J Cancer 91, 225-32[46]	2004	385	0.001

Aksnes et al, Acta Oncol 45, 38-46[30]	2006	56	0.001

Kreuter, Eur J Cancer 45, 1904-11[47]	2006	40	S

La et al, Int J Radiat Oncol Biol Phys 64 (2), 544-50[48]	2006	60	0.036

Cheung et al, Clin Cancer Res 13 (23), 6978-83[32]	2007	28	0.04

Leavey et al, Pediatr blood Cancer 51 (3), 334-8[18]	2008	262	0.02

Yonemori et al, J Cancer Res Clin Oncol 134, 389-95[34]	2008	79	0.02

Jawad et al, Cancer 115, 3526-36[19]	2009	385	0.001

Sari et al, Pediatr Blood Cancer 54, 19-24[21]	2010	87	0.001

Xie et al, Chin J Cancer 29 (4), 420-4	2010	18	0.01

NS: not significant, S: significant

**Table 4:** Outcome for phenotypic marker: histological response

Author	Year	Pt number	P
Delepine et al, J Chemother 9 (5), 352-63[49]	1997	39	0.05

Picci et al, J Clin Oncol 15 (4), 1553-9[50]	1997	118	0.0001

Aparicio et al, Oncology 55, 20-6[9]	1998	116	0.018

Paulussen et al, J Clin Oncol 16 (9), 3044-52[39]	1998	114	S

Abudu et al, J Bone Joint Surg 81 (2), 317-22[51]	1999	50	0.03

Ahrens et al, Med Pediatr Oncol 32, 186-95[10]	1999	177	0.27

Baldini et al, Ann Surg 230 (1), 79-86[40]	1999	37	0.01

Bacci et al, J Clin Oncol 18, 4-11[13]	2000	359	0.001

De Alava et al, Cancer 89, 783-92[25]	2000	55	0.001

Ohali et al, J Clin Oncol 21, 3836-43[52]	2003	31	0.0001

Scotlandi et al, Eur J Cancer 41, 1349-61[53]	2005	113	0.05

Bacci et al, Acta Oncol 45, 469-75[16]	2006	579	0.0005

Mikulic et al, J Pediatr Surg 41, 524-9[31]	2006	27	0.047

Avigad et al, Clin Cancer Res 13 (19), 5777-83[54]	2007	32	0.13

Yonemori, J Cancer Res Clin Oncol 134, 389-95v [34]	2008	79	0.04

Meynet et al, Cancer Res 70 (9), 3730-8[55]	2010	97	0.02

S: significant

### Primary outcome

The investigated biomarkers are subdivided in two groups, phenotypic markers and biological markers. For the phenotypic markers gender, tumour size, metastases and histological response are reported in Tables 1, 2, 3 and 4. For all these phenotypic markers we compared the patient number and p-value, in which p < 0.05 was taken as statistically significant. However we weren't able to retrieve the p-value in all articles, sometimes it was only mentioned as being significant or non-significant. For each phenotypic marker we looked at the differences in overall survival between: for gender, men vs women; for tumour size, < 8-10 cm vs > 8- 10 cm; for metastasis presence at initial presentation vs absence and for histological response, > 90% necrosis vs < 90% necrosis. Distributions of p related to patient numbers in these four phenotypic markers are shown in Figures 2. For these four phenotypic markers we show that there is no correlation between the number of patients and the statistical significance of the outcome. More phenotypic markers were reported: fusion type, ethnicity, performance status and margins. However because of the low number of studies which reported these outcomes these results are not shown in detail. In 26 articles the impact of tumour site on the overall survival is shown, but because sites are compared in different ways it is difficult to summarize these findings.

Currently clinical stage is being used to determine whether a patient has a high or low risk for developing metastases or recurrence. However, it seems that clinical stage is not always related to outcome, because of the patients who present with non-metastatic disease, only 70% of them survive for 5-years. Therefore, what is the difference between the 70% of the patients who survive and the 30% who don't? Can one somehow foretell chemotherapy resistance and detect metastases early? One way to predict the outcome of patients apart from clinical stage is to use biomarkers. These are objective measurements which reflect biological processes. The biomarkers currently being used are tumour size and the presence of metastases. Biological markers are not being used, even though they may provide a way to predict a patient's outcome more accurately than phenotypic markers. From the results for phenotypic markers we can see that gender is probably not significant important for patient outcome. In 15 articles we found 11 reports that gender is non-significant. Tumour size > 8 cm seems to be important, with 15 out of 22 articles finding it to be a predictor and significantly related to negative outcome. The presence of metastasis is a strong predictor of negative outcome with 24 articles reporting it as significantly relevant compared to only 3 reporting it as non-significant. For histological response, 12 out of 16 articles found that necrosis > 90% after treatment is a significant predictor for positive outcome.

For some phenotypic markers it is unclear how the cut-off point between predictor of positive or negative outcomes is determined. For tumour size the cut-off point for negative outcome is > 8 cm, but it is undefined how this is selected. It seems more logical that tumour size is a continuous variable with an increasingly negative outcome with increasing size. The same can probably be said for age and surgical margins.

**Figure 3:**
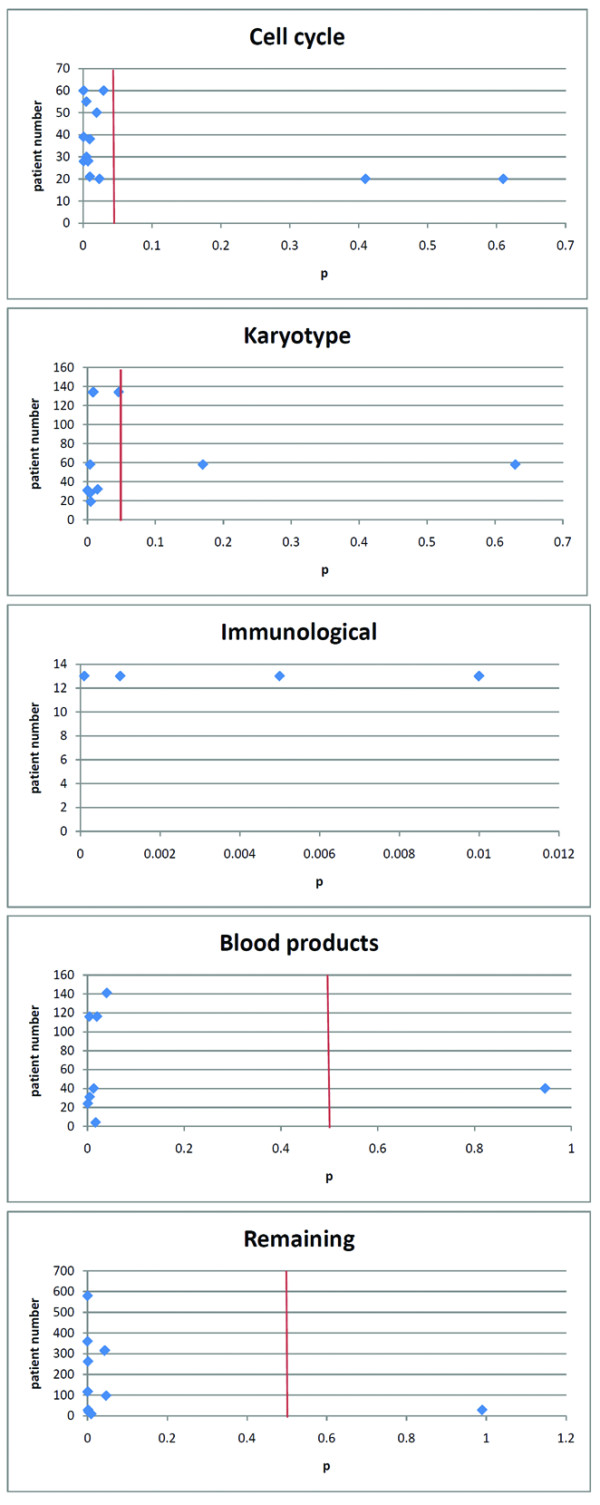
**Distribution of p related to patient number for the biological markers related to cell cycle, karyotype, immunological, blood products and remaining markers**. The red line shows the cut-off point of p = 0.05. Note, there is no line for immunological phenotypic markers because for all the results p < 0.05.

Biological markers are more difficult to compare, because for most of these markers only one or two reports are published. We grouped the biological markers according to their function and we ended up with 5 groups, namely cell cycle, karyotype, immunological, blood products and the remaining biological markers which couldn't be classified in one of the other groups. The results from the biological markers are shown in Tables 5, 6, 7, 8 and 9. The correlation between patient number and statistical significance of the outcome for the five groups is shown in Figures 3. We show that there is no correlation between the patient number and the statistical significance of the outcome. It appears that ki67, an S-phase cell cycle biomarker, may be a biomarker of cell activity in the tumour that significantly correlates with outcome. The mechanism for the activation of cell cycle appears unclear, but is presumably driven by other factors other that EWS-FLI1 translocation. Loss of function of cell cycle dependent kinases (p16, p14, p21) and other regulators of the cell cycle through the p53 pathway (MDM2, p53), also appear deregulate in a proportion of tumours and potentially are useful prognostic markers. Importantly, activity of telomerase appears significantly correlated with outcome as occurs in many other tumours. There appears much interest in secondary copy number changes and mutations in Ewing sarcoma, and in particular, chromosome 1 (Table 6). For example, recent evidence points to gain of 1q and alteration in abundance of a gene product called CDt2 involved in ubiquitination [7]. It is however difficult to objectively say anything about the other reported markers because they may influence each other. This appears most clear for tumour size and metastases, where bigger tumours may correlate with a higher chance of having metastases. For biological markers it is probably the same issue, but less clear because we don't really know their true experimental influence on tumour genesis. For example, LDH levels are probably a reflection of cell turnover in larger tumours, and may be an indirect measure of bulk of disease (comparing Table 2 versus Table 9). It is also more difficult to say anything about biological markers because they haven't been tested as extensively as phenotypic markers, and certainly they have not often been validated independently. Results for most of these markers are only reported in 1 or 2 articles with sometimes small numbers of patients and no statistical validation. To improve this situation it would important to capture high quality clinical material and clinical outcome to develop a bio-bank. We may be able to test the most promising biomarkers from previously run studies and so define their significance. Either a multivariate analysis or data mining analysis should be done to evaluate the way biomarkers affect each other. The easiest way to achieve this objective is by collecting material and outcome data from large phase III trials. It is also important to standardize the way material is collected and how the biomarkers are compared. For example, the phenotypic marker tumour site is the most often tested marker with results published in 26 articles (data not shown). However it is not possible to say anything about these results since different tumour sites are compared in the reports. This is also true for the marker age in which different age groups are compared with each other, for example some articles compare patients < 18 years vs > 18 years, others < 30 years vs > 30 years (data not shown).

**Table 5:** Outcome for biological markers: cell cycle

Author	Year	Biomarker	Pt number	P
Landanyi et al, J Pathol 175 (2), 211-7	1995	MDM-2	30	0.005

Luksch et al, Tumori 85 (2), 101-7[41]	1999	Mitose presence	73	S

Sollazzo et al, tumori 85 (3), 167-73[56]	1999	Ki-67	38	0.01

De Alava et al, Cancer 89, 783-92[25]	2000	Ki-67	55	0.005

Abudu et al, Br J Cancer 79(7-8), 1185-9[57]	1999	P53	50	0.02

Huang et al, J Clin Oncol 23, 548-58[58]	2005	P53	60	0.001

Matsunobu et al, C;in Cancer Res 10, 1003-12[29]	2004	P27	21	0.01

Wei et al, Cancer 89, 793-9[42]	2000	INK4a	39	0.001

Maitra et al, Arch Pathol Lab Med 125, 1207-12[59]	2001	P16INK4a	20	0.41

Maitra et al, Arch Pathol Lab Med 125, 1207-12[59]	2001	P14ARF	20	NS

Huang et al, J Clin Oncol 23, 548-58[58]	2005	P16/p14ARF	60	0.03

Maitra et al, Arch Pathol Lab Med 125, 1207-12[59]	2001	P21WAF1	20	0.61

Ohali et al, Oncogene 23, 8997-9006[60]	2004	Cadherin-11	20	0.024

Cheung et al, Clin Cancer Res 13 (23), 6978-83[32]	2007	STEAP1	28	0.0012

Cheung et al, Clin Cancer Res 13 (23), 6978-83[32]	2007	CCND1	28	0.0077

Martins et al, Cancer Res 68 (15), 6260-70[61]	2008	Heat shock 90	54	S

Zanini et al, Virchows Arch 452, 157-67[62]	2008	Heat shock 27	unknown	NS

S: significant, NS: not significant

**Table 6:** Outcome for biological markers: karyotype

Author	Year	Biomarker	Pt number	P
Tarkannen et al, Cancer Genet Cytogenet 114, 35-41	1999	1q	28	NS

Hattinger et al, Br J Cancer 86, 1763-9[63]	2002	1q	134	0.046

Tarkannen et al, Cancer Genet Cytogenet 114, 35-41	1999	6p2.1	28	0.004

Lopez-Guerrero et al, Lab Invest 81 (6), 803-14[64]	2001	9p21 locus	19	0.005

Hattinger et al, Br J Cancer 86, 1763-9[63]	2002	16q	134	0.008

Hattinger et al, Genes Chromosomes Cancer 24 (3), 243-54[65]	1999	Chr 1	58	0.004

Tarkannen et al, Cancer Genet Cytogenet 114, 35-41	1999	Chr 8	28	NS

Hattinger et al, Genes Chromosomes Cancer 24 (3), 243-54[65]	1999	Chr 8	58	0.17

Hattinger et al, Br J Cancer 86, 1763-9[63]	2002	Chr 8	134	NS

Tarkannen et al, Cancer Genet Cytogenet 114, 35-41	1999	Chr 12	28	NS

Hattinger et al, Genes Chromosomes Cancer 24 (3), 243-54 [65]	1999	Chr 12	58	0.63

Hattinger et al, Br J Cancer 86, 1763-9[63]	2002	Chr 12	134	0.009

Ohali et al, J Clin Oncol 21, 3836-43[52]	2003	Telomerase activity	31	0.0001

Avigad et al, Clin Cancer Res 13 (19), 5777-83[54]	2007	Telomerase length	32	0.015

NS: not significant, Chr: Chromosome

**Table 7:** Outcome for biological markers: immunological

Author	Year	Biomarker	Pt number	P
Rutkowski et al, J Surg Oncol 84, 151-9[27]	2003	IL-1ra	13	0.0001

Rutkowski et al, J Surg Oncol 84, 151-9[27]	2003	sIL-2ra	13	0.005

Rutkowski et al, J Surg Oncol 84, 151-9[27]	2003	IL-6	13	0.001

Rutkowski et al, J Surg Oncol 84, 151-9[27]	2003	IL-8	13	0.0001

Rutkowski et al, J Surg Oncol 84, 151-9[27]	2003	IL-10	13	0.01

Rutkowski et al, J Surg Oncol 84, 151-9[27]	2003	TNF RI	13	0.001

Rutkowski et al, J Surg Oncol 84, 151-9[27]	2003	TNF RII	13	0.01

Rutkowski et al, J Surg Oncol 84, 151-9[27]	2003	M-CSF	13	0.01

Berghuis et al, J Pathol 218, 222-31[66]	2009	HLA class I	67	NS

NS: not significant

**Table 8:** Outcome for biological markers: blood products

Author	Year	Biomarker	Pt number	P
Holzer et al, Med Pediatr Oncol 36 (6), 601-4[67]	2001	VEGF	6	NS

Pavlakovic et al, Int J Cancer 92, 756-60 [68]	2001	VEGF	4	0.017

Rutkowski et al, J Surg Oncol 84, 151-9[27]	2003	VEGF	13	NS

Fuchs et al, Clin Cancer Res 10, 1344-53[45]	2004	VEGF	31	0.0047

Jimeno et al, Pediatr Blood Cancer 49, 352-7[69]	2007	VEGF	16	NS

Kreuter et al, Eur J Cancer 42, 1904-11[47]	2006	VEGF-A	40	0.013

Kreuter et al, Eur J Cancer 42, 1904-11[47]	2006	VEGFR-1	40	0.946

Kreuter et al, Eur J Cancer 42, 1904-11[47]	2006	VEGFR-2	40	0.946

Aparicio et al, Oncology 55, 20-6[9]	1998	Lymphocyte count	116	0.0044

De Angulo et al, J Pediatr Hematol Oncol 29 (1), 48-52[17]	2007	Lymphocyte count	24	0.001

De Angulo et al, J Pediatr Hematol Oncol 29 (1), 48-52 [17]	2007	Platelet count	24	NS

De Angulo et al, J Pediatr Hematol Oncol 29 (1), 48-52[17]	2007	Neutrophil count	24	NS

Aparicio et al, Oncology 55, 20-6[9]	1998	Erythrocyte sedimentation rate	116	0.02

Oberlin et al, B J Cancer 85 (11), 1646-54[26]	2001	Erythrocyte sedimentation rate	141	0.04

Yabe et al, Oncol Rep 19 (1), 129-34[70]	2008	Erythrocyte sedimentation rate	20	NS

NS: not significant

**Table 9:** Outcome for biological markers: remaining

Author	Year	Biomarker	Pt number	P
Craft et al, Eur J Cancer 33 (7), 1061-9[8]	1997	LDH	142	NS

Aparicio et al, Oncology 55, 20-6[9]	1998	LDH	116	0.001

Givens et al, Int J Oncol 14 (6), 1039-43[12]	1999	LDH	85	NS

Bacci et al, Oncol Rep 6 (4), 807-11[71]	1999	LDH	618	S

Luksch et al, Tumori 85 (2), 101-7[41]	1999	LDH	73	S

Bacci et al, J Clin Oncol 18, 4-11[13]	2000	LDH	359	0.0003

Matsunobu et al, Clin Cancer Res 10, 1003-12[29]	2004	LDH	21	NS

Bacci et al, Acta Oncol 45, 469-75[16]	2006	LDH	579	0.0005

Cheung et al, Clin Cancer Res 13 (23), 6978-83[32]	2007	LDH	28	0.99

Yabe et al, Oncol Rep 19 (1), 129-34[70]	2008	LDH	20	NS

Leavey et al, Pediatr Blood Cancer 51 (3), 334-8[18]	2008	LDH	262	0.0016

Xie et al, Chin J Cancer 29 (4), 420-4	2010	LDH	18	NS

Terrier et al, Eur J Cancer 31 (3), 307-14[36]	1995	Filigree pattern	315	0.044

Terrier et al, Eur J Cancer 31 (3), 307-14[36]	1995	Dark cells	315	0.043

Aparicio et al, Oncology 55, 20-6[9]	1998	Albumine levels	116	0.0006

Sollazzo et al, Tumori 85 (3), 167-73[56]	1999	c-myc	38	S

Ohali et al, Oncogene 23, 8997-9006[60]	2004	MTA1	20	0.003

Cheung et al, Clin Cancer Res 13 (23), 6978-83[32]	2007	NKX2-2	28	0.0017

Kikuta et al, Clin Cancer Res 15 (8), 2885-94[20]	2009	Nucleophosmin positivity	8	0.01

Meynet et al, Cancer Res 70 (9), 3730-8[55]	2010	Xg expression	97	0.047

S: significant, NS: not significant

For markers of tumour growth, angiogenesis if often quantified, but so far biomarker analysis has been predominantly limited to measurement of VEGF pathway (Table 8). The immunological biological markers interleukins and tumour necrosis factors seem very promising (Table 7). However these have all been tested in one institute, with very small patient numbers and the data doesn't seem to be validated. Most of the biological markers mentioned in the blood products group (Table 8) are probably surrogates for tumour size and they should be validated in either a multivariate analysis or machine learning to see if they can be used as an objective biological marker.

At the present time it is no possible to make a definite list of biological biomarkers able to predict patient outcome, mainly because these markers also have to be stratified with respect to the major staging phenotypic features, e.g. presence of metastasis and degree of histological response. It is also unclear what quality control measure were used in the limited patient cohorts. Our recommendation would be continue divide patients according to their disease stage and also to use the phenotypic biomarkers metastasis, tumour size and histological response. For biological biomarkers we would like to validate previous work done on the markers for 9p21 locus and the involved genes and proteins, heat shock proteins, telomerase related markers, interleukins, tumour necrosis factors, VEGF pathway, lymphocyte count, MTA1, STEAP1, CCND1, MDM-2, Ki-67, p53, p27 and cadherin-11. At this time, neither phenotypic (clinical) or biological biomarkers are utilised in stratification of patients in clinical trials.

## Lists of abbreviations

LDH: Lactate dehydrogenase; REMARK: Reporting recommendations for tumour MARKer prognostic studies; ESFT: Sarcoma Family of Tumours; PNET: Primitive Neuroectodermal.

## Competing interests

All authors have no competing financial interests in the publication of this manuscript.

No organisation is funding or implicated in the manuscripts analysis and interpretation.

Academic interests of the authors are to improve the outcome of patients with sarcoma, and this publication forms part of the deliverable output from EU funding from EuroBoNeT.

## Authors' contributions

ABH conceived the study, AVM collected data with ABH, AVM and ABH wrote the paper and PCH made detailed comments. All authors have read and approved the final version of the manuscript.
